# Sex-based analysis of NSTEMI processes of care and outcomes by hospital: a nationwide cohort study

**DOI:** 10.1093/ehjqcco/qcae011

**Published:** 2024-02-06

**Authors:** Nicholas Weight, Saadiq Moledina, Evangelos Kontopantelis, Harriette Van Spall, Mohammed Dafaalla, Alaide Chieffo, Mario Iannaccone, Denis Chen, Muhammad Rashid, Josepa Mauri-Ferre, Jacqueline E Tamis-Holland, Mamas A Mamas

**Affiliations:** Keele Cardiovascular Research Group, Centre for Prognosis Research, Institute for Primary Care and Health Sciences, Keele University, Stoke-on-Trent, ST5 5BG, UK; Keele Cardiovascular Research Group, Centre for Prognosis Research, Institute for Primary Care and Health Sciences, Keele University, Stoke-on-Trent, ST5 5BG, UK; Division of Informatics, Imaging and Data Sciences, University of Manchester, Manchester, M1 3BB, UK; Department of Medicine, McMaster University, Hamilton, Ontario L8N 3Z5, Canada; Department of Health Research Methods, Evidence, and Impact, McMaster University, Hamilton, Ontario, Canada; Population Health Research Institute, Hamilton, Ontario L8L 2X2, Canada; Keele Cardiovascular Research Group, Centre for Prognosis Research, Institute for Primary Care and Health Sciences, Keele University, Stoke-on-Trent, ST5 5BG, UK; Interventional Cardiology Unit, IRCCS San Raffaele Hospital, Milan, Via Olgettina 60 20132, Italy; Division of Cardiology, San Giovanni Bosco Hospital, Turin 10154, Italy; Keele Cardiovascular Research Group, Centre for Prognosis Research, Institute for Primary Care and Health Sciences, Keele University, Stoke-on-Trent, ST5 5BG, UK; Keele Cardiovascular Research Group, Centre for Prognosis Research, Institute for Primary Care and Health Sciences, Keele University, Stoke-on-Trent, ST5 5BG, UK; Department of Cardiovascular Sciences, University of Leicester, Leicester, LE1 7RH, UK; NIHR Leicester Biomedical Research Centre, Glenfield Hospital, University Hospitals of Leicester NHS Trust, Leicester, LE1 5WW, UK; Departament de Salut, Gobierno de Cataluña, Barcelona 08028, Spain; Servicio de Cardiología, Hospital Universitario Germans Trias i Pujol, Badalona, Barcelona 08916, Spain; Cardiovascular Institute, Icahn School of Medicine at Mount Sinai, New York, NY 10029, USA; Keele Cardiovascular Research Group, Centre for Prognosis Research, Institute for Primary Care and Health Sciences, Keele University, Stoke-on-Trent, ST5 5BG, UK

**Keywords:** Sex, Quality of care, NSTEMI

## Abstract

**Background:**

Contemporary studies demonstrate that non-ST-segment elevation myocardial infarction (NSTEMI) processes of care vary according to sex. Little is known regarding variation in practice between geographical areas and centres.

**Methods:**

We identified 305 014 NSTEMI admissions in the United Kingdom (UK) Myocardial Ischaemia National Audit Project (MINAP), 2010–17, including female sex (110 209). Hierarchical, multivariate logistic regression models were fitted, assessing for differences in primary outcomes according to sex. Risk-standardized mortality rates (RSMR) were calculated for individual hospitals to illustrate the correlation with variables of interest. ‘Heat maps’ were plotted to show regional and sex-based variation in the opportunity-based quality indicator score (surrogate for optimal processes of care).

**Results:**

Women presented older (77 years vs. 69 years, *P* < 0.001) and were more often Caucasian (93% vs. 91%, *P* < 0.001). Women were less frequently managed with an invasive coronary angiogram (58% vs. 75%, *P* < 0.001) or percutaneous coronary intervention (35% vs. 49%, *P* < 0.001). In our hospital-clustered analysis, we show a positive correlation between the RSMR and the increasing proportion of women treated for NSTEMI (*R*^2^ = 0.17, *P* < 0.001). There was a clear negative correlation between the proportion of women who had an optimum OBQI score during their admission and RSMR (*R*^2^ = 0.22, *P* < 0.001), with a weaker correlation in men (*R*^2^ = 0.08, *P* < 0.001). Heat maps according to the Clinical Commissioning Group (CCG) demonstrate significant regional variation in the OBQI score, with women receiving poorer quality care throughout the UK.

**Conclusion:**

There was a significant variation in the management of patients with NSTEMI according to sex, with widespread geographical variation. Structural changes are required to enable improved care for women.

## Graphical Abstract

**Graphical Abstract:**
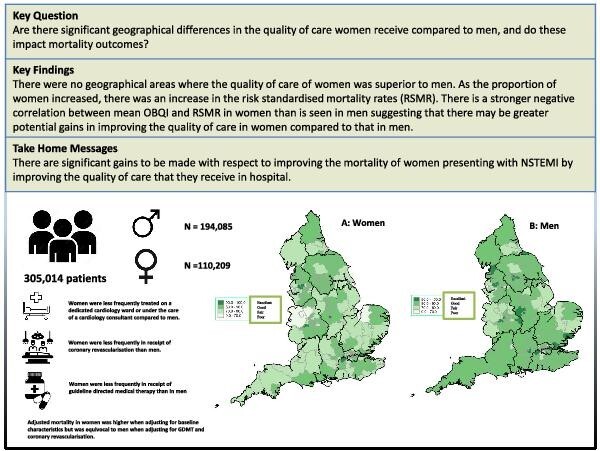

Key learning points **What is already known**

- There are sex-based differences in the processes of care and in-hospital outcomes following acute myocardial infarction.
- Women typically receive poorer quality care, with respect to prescription of guideline directed medical therapy and invasive investigations and revascularization.
- Regional differences in quality of care have been reported in the United States, but less is known regarding whether this is evident in the UK, and whether this varies according to sex.

**Key Findings:**

- Women had poorer quality care in all regions of the UK, and there was significant regional variation in quality of NSTEMI care throughout the UK.
- The correlation between quality of care and mortality is stronger in women than in men.
- Improving the quality of NSTEMI care in women to the same standard of men UK-wide would have a significant impact on UK NSTEMI mortality.

## Introduction

Cardiovascular disease is the leading cause of death in women in Europe and worldwide,^1^ accounting for greater than half of all female deaths in Europe, and has worse outcomes and higher mortality than in men.^2^ Despite this, contemporary studies have demonstrated that in acute myocardial infarction (AMI), processes of care vary according to sex. Women have been demonstrated to be less frequently prescribed guideline-directed medical therapy (GDMT), less likely to be investigated by or receive timely invasive coronary angiography (ICA), and less likely to be treated with percutaneous coronary intervention (PCI) or coronary artery bypass graft surgery (CABG).^3–8^

Whilst sex disparities in AMI outcomes have been studied extensively, there is limited information regarding whether there is significant variation in practice between geographical areas and individual centres and whether this impacts clinical outcomes. Thus, in this large national European study, we describe sex differences in the clinical characteristics, management strategies, and outcomes of patients presenting with non-ST-segment elevation myocardial infarction (NSTEMI) and explore regional/inter-hospital variations in the quality of care received by women. Finally, we aim to study whether there are sex differences in the association between the quality of NSTEMI care and clinical outcomes.

## Methods

### Study design

We used the Myocardial Ischaemia National Audit Project (MINAP), a prospective national clinical registry of patients admitted to hospitals in the UK with acute coronary syndrome.^9^ The MINAP dataset consists of 130 variables, including baseline demographics and clinical characteristics, comorbidities, management strategies, pharmacotherapy, in-hospital clinical outcomes, and discharge diagnosis,^10^ and there is a minimum standard for the data-completeness of submitted records, which is 95% completion of 43 key variables for NSTEMI patients. Data are submitted by hospital clinical and clerical staff, and approximately 90 000 pseudonymized records annually are uploaded to the National Institute for Cardiovascular Outcomes Research (NICOR). Patient data is collected up to the point of hospital discharge, without external adjudication of study endpoints. There is annual data validation at each participating hospital using randomly selected patient records and re-entry of data.^11^

### Study population

**Figure 1:**
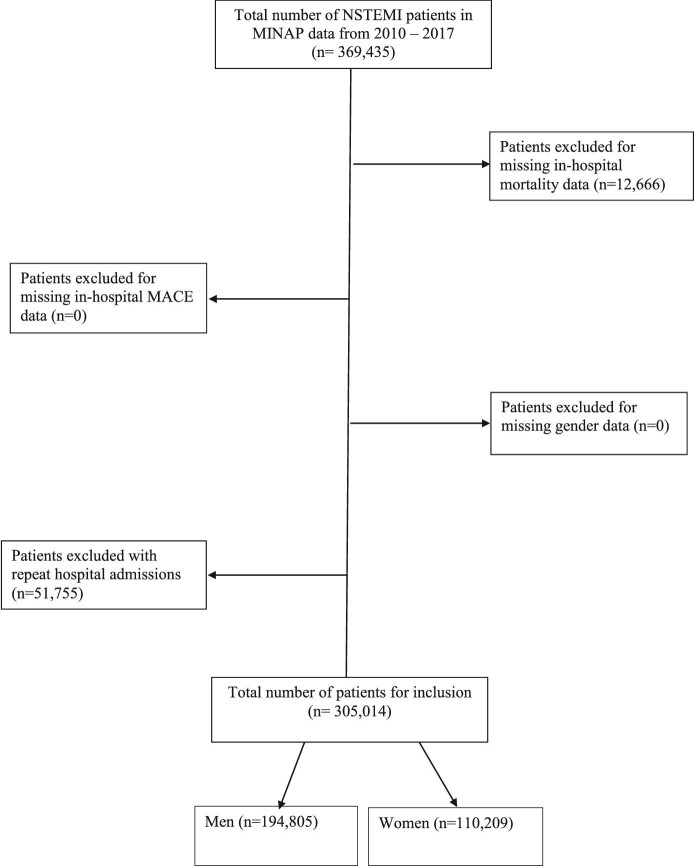
STROBE diagram detailing exclusion criteria.

We included patients admitted with a diagnosis of NSTEMI in any of the 230 participating hospitals in England and Wales between 1 January 2010 and 31 March 2017. The discharge diagnosis of NSTEMI was determined by local clinicians according to presenting history, clinical examination, and the results of inpatient investigations in keeping with the consensus document of the Joint European Society of Cardiology (ESC) and American College of Cardiology.^12^ Patients were excluded if they had missing data in our key variables for investigation: sex, in-hospital mortality, and major adverse cardiovascular events (MACE). Furthermore, any individual patient's subsequent admissions were excluded from analysis (*Figure 1*).

### Outcomes

#### Primary

Primary outcomes of interest included in-hospital all-cause mortality and MACE (composite endpoints of in-hospital mortality and reinfarction).

#### Secondary

Secondary outcomes of interest included cardiac mortality [death attributable to myocardial ischaemia or infarction, heart failure, and cardiac arrest of unknown cause] and major bleeding.

### Quality indicators

We assessed the (ESC) Association for Acute Cardiovascular Care (ACVC) quality indicators (QI's),^13^ including the use of ICA within 72 h of admission; the assessment of left ventricular (LV) function while an inpatient; the use of fondaparinux or low molecular weight heparin (LMWH); and the prescription of P2Y_12_ inhibition, adequate dual antiplatelet therapy (DAPT), and statins on discharge. For patients with moderate and severe LV systolic dysfunction (LVSD), the use of angiotensin converting enzyme inhibitors (ACEi) or angiotensin receptor blockers (ARB) and beta blockers on discharge was also evaluated. The ESC QI for LVSD is defined as an ejection fraction (EF) less than or equal to 40%. The MINAP database does not have the same cut-off points for LVSD, thus moderate (EF <49%) and severe LVSD (EF <30%) were used as surrogates. Furthermore, MINAP does not record the specific type or dose of statin prescribed, so ‘statin prescription’ was used as a surrogate for high-intensity statin.

### Statistical analysis

Demographics, clinical characteristics, and crude adverse outcomes of patients by sex were compared using the Pearson chi-squared test for categorical variables. Continuous variables were compared using the Student's *t*-test if normally distributed and using the Wilcoxon rank sum test if not. Normality of the distribution was assessed using the Shapiro-Wilks test. Continuous variables are presented as medians and interquartile ranges (IQR), and categorical variables by proportions. Multiple imputations with chained equations (MICE) were used to impute values for variables with missing data. MICE is considered to be the best practice when dealing with missing data and can provide unbiased estimates even when levels of missing data are significant, and also some protection when the pattern of ‘missingness’ is not random.^14^ For each binary outcome of interest, multivariable logistic regression analysis was applied to imputed datasets to estimate the risk of adverse outcomes between groups. Estimates were combined using Rubin's rules.^15^ Hierarchical logistic regression models with patients nested within hospitals using maximum likelihood estimation were adjusted for: age, ethnicity, creatinine, heart rate and systolic blood pressure on admission, history of angina, family history of coronary artery disease, co-morbid conditions (hypertension, hypercholesterolaemia, diabetes, smoking and history of asthma or COPD), pharmacotherapy [prescription of LMWH, unfractionated heparin (UFH), warfarin, GP 2b/3a inhibitor, IV nitrate, furosemide, aldosterone antagonist, fondaparinux, beta blockers, ACEi/ARBs, aspirin, P2Y12 inhibitor statins, thiazide diuretics], cardiac arrest, and procedures including ICA and revascularization (by PCI or CABG) during admission. A second model assessing the interaction between the Opportunity Based Quality Indicator (OBQI) score category and sex was performed, adjusting with the aforementioned variables. OBQI score was ordered into four categories, ‘Excellent’, ‘Good’, ‘Fair’, and ‘Poor’. Excellent refers to an OBQI score of ≥90 and ≤100, Good a score of ≥80 and <90, Fair ≥70 and <80, and Poor <70. The difference in adjusted mortality between categories of OBQI score was used to multiply the number of female patients per region to estimate the potential number of lives saved if women in that region received the same category of care as men.

### Temporal and geographical changes

Risk-standardized mortality rates (RSMR), adjusted for patient demographics, were calculated for each hospital included in our study. The mortality assessed was in-hospital mortality only. We undertook logistic regression to ascertain whether there was a correlation with the adjusted mortality rates and proportion of patients that were female. The OBQI is a composite score comprising the prescription of aspirin, thienopyridine inhibitor, β-blocker, ACEi, HMG CoA reductase enzyme inhibitor (statin), and enrolment onto a cardiac rehabilitation programme at the time of discharge. The OBQI reflects the number of care opportunities fulfilled at each hospital (numerator) divided by the number of opportunities to provide care (denominator). Excluded from both the numerator and denominator were interventions that were contra-indicated, not applicable, not indicated in, or declined by, individual patients. We formed scatterplots to assess the correlation of RSMR for each hospital with the mean OBQI score for each sex. We created ‘heat maps’ according to mean the OBQI according to 2021 England and Wales Clinical Commissioning Group regions (CCGs), clustering hospitals together according to 2021 CCG geographical boundaries. ‘Heat maps’ were formed for the overall mean OBQI for each CCG and for women and men separately.

Statistical analysis was undertaken using Stata 14.2 (College Station, Texas, USA). All statistical analyses were two-tailed, with an alpha of 5% used.

## Results

### Demographics

During the study period, there were 369 435 patients admitted to England and Wales with a diagnosis of NSTEMI. Applying the exclusion criterion left an analytical cohort of 305 014 patients, of which 110 209 were women (36%) (*Figure 1*). The differences in clinical characteristics between the two groups are presented in *Table 1*. Women were significantly older at presentation [(77 years old (IQR 67–85) vs. 69 years old (IQR 59–79), *P* < 0.001], less frequently from an ethnic minority background (7% vs. 9%, *P* < 0.001), less frequently had a previous history of smoking (29% vs. 41%, *P* < 0.001) or current smoking (18% vs. 24%, *P* < 0.001), were less likely to be admitted under a consultant cardiologist (44% vs. 52%, *P* < 0.001) or admitted to a dedicated cardiology ward (48% vs. 56%, *P* < 0.001), and more frequently presented with a ‘High-risk’ GRACE score (>140) (82% vs. 71%, *P* < 0.001) than men.

**Table 1:** Demographic comparison between male and female NSTEMI patients

Variable	Males (*n* = 194 805)	Females (*n* = 110 209)	*P*-value
Age, years, median (IQR)	69 (59–79)	77 (67–85)	<0.001
BMI, median [IQR]	27.5 (24.6–30.8)	26.4 (23.0–30.8)	<0.001
Ethnicity-White	162 294/177 662 (91)	94 673/101 262 (93)	<0.001
Ethnicity—Ethnic minority	15 368/177 662 (9)	6 589/101 262 (7)	<0.001
Killip class			
Basal crepitations (%)	18 409/125 417 (15)	13 550/69 520 (19)	<0.001
Pulmonary oedema (%)	6 653/125 417 (5)	5 326/69 520 (8)	<0.001
Cardiogenic shock (%)	752/125 417 (1)	418/69 520 (1)	<0.001
GRACE—risk score			
High risk GRACE score >140 (%)	86 148/121 545 (71)	55 256/67 512 (82)	<0.001
Intermediate risk GRACE score 109–140 (%)	26 157/121 545 (22)	9 593/67 512 (14)	<0.001
Low risk GRACE score <109 (%)	9 240/121 545 (8)	2 663/67 512 (4)	<0.001
ECG ST changes (%)	140 343/189 501 (74)	82 718/107 263 (77)	<0.001
Comorbidities			
Previous smoker (%)	76 811/185 667 (41)	29 801/103 264 (29)	<0.001
Current smoker (%)	45 276/185 667 (24)	18 411/103 264 (18)	<0.001
Chronic renal failure (%)	15 592/182 675 (9)	9 638/103 385 (9)	<0.001
Prior percutaneous coronary intervention (%)	27 571/182 738 (15)	10 027/103 374 (10)	<0.001
Diabetes (%)	49 653/191 958 (26)	27 277/108 592 (25)	<0.001
CCF (%)	12 987/182 705 (7)	9 219/103 278 (9)	<0.001
Hypercholesterolaemia (%)	66 768/181 581 (37)	33 607/102 699 (33)	<0.001
Previous MI (%)	54 249/184 411 (29)	25 211/104 230 (24)	<0.001
Cerebrovascular disease (%)	17 295/183 043 (9)	11 898/103 555 (11)	<0.001
History of angina (%)	55 909/182 563 (31)	29 873/103 327 (29)	<0.001
Peripheral vascular disease (%)	10 720/182 195 (6)	4 634/103 070 (5)	<0.001
Hypertension (%)	97 228/184 313 (53)	61 786/104 416 (59)	<0.001
Asthma/COPD (%)	29 172/183 177 (16)	21 426/103 689 (21)	<0.001
Family history of CAD (%)	45 347/154 910 (29)	21 122/85 173 (25)	<0.001
Heart rate, bpm, median (IQR)	76 (65–90)	81 (70–96)	<0.001
Systolic blood pressure, median (IQR)	138 (121–156)	141 (122–161)	<0.001
LV function			
Normal LV function (%)	58 302/98 132 (59)	32 661/52 931 (62)	<0.001
Moderate LVSD (%)	27 680/98 132 (28)	14 616/52 931 (28)	<0.001
Severe LVSD (%)	12 150/98 132 (12)	5 654/52 931 (11)	<0.001
Cardiac arrest (%)	6 698/190 620 (4)	3 418/107 912 (3)	<0.001
Previous CABG surgery (%)	19 963/183 014 (11)	5 232/103 510 (5)	<0.001
Admission under cardiologist (%)	95 405/183 868 (52)	45 245/102 964 (44)	<0.001
Admission to cardiology ward (%)	108 976/193 583 (56)	52 598/109 560 (48)	<0.001

CABG, coronary artery bypass graft; LVSD, left ventricular systolic dysfunction; CAD, coronary artery disease; COPD, chronic obstructive pulmonary disease; MI, myocardial infarction; CCF, congestive cardiac failure; BMI, body mass index; GRACE, global registry of acute coronary events; IQR, interquartile range. Admission to cardiology ward is a composite of admission to coronary care unit (CCU) and general cardiology ward.

### Management and clinical outcomes

Pharmacotherapies, management strategies, and unadjusted crude clinical outcomes for both cohorts are presented in *Table 2*. Women were less frequently prescribed statins (80% vs. 84%, *P* < 0.001), ACEi or ARBs (70% vs. 74%, *P* < 0.001), beta-blockers (79% vs. 82%, *P* < 0.001), and P2Y12 inhibitors (90% vs. 92%, *P* < 0.001) (*Table 2*). They less frequently underwent ICA (58% vs. 75%, *P* < 0.001), PCI (35% vs. 49%, *P* < 0.001), or CABG surgery (5% vs. 9%, *P* < 0.001) during their index NSTEMI admission. Women had a higher frequency of in-hospital mortality (7% vs. 5%, *P* < 0.001), MACE (7% vs. 5%, *P* < 0.001), cardiac mortality (5% vs. 4%, *P* < 0.001), and major bleeding (2% vs. 1%, *P* < 0.001) when compared to men in our unadjusted data.

**Table 2:** Sex differences in management strategy and clinical outcomes in NSTEMI

Variables	Males (*n* = 194 805)	Females (*n* = 110 209)	*P*-value
Low molecular weight heparin (%)	85 909/166 581 (52)	50 410/94 999 (53)	<0.001
Fondaparinux (%)	78 834/167 203 (47)	44 461/95 399 (47)	0.007
Warfarin (%)	10 466/165 574 (6)	6338/94 586 (7)	<0.001
Unfractionated heparin (%)	25 305/165 001 (15)	10 659/94 286 (11)	<0.001
Glycoprotein 2b/3a inhibitor (%)	5945/167 937 (4)	2226/95 723 (2)	<0.001
IV Nitrate (%)	21 740/165 502 (13)	11 395/94 606 (12)	<0.001
Furosemide (%)	42 137/165 933 (25)	32 854/94 898 (35)	<0.001
Calcium channel blockers (%)	31 070/165 694 (19)	19 070/94 689 (20)	<0.001
IV beta blockers (%)	1888/166 452 (1)	1138/94 983 (1)	0.142
MRA (%)	11 403/164 515 (7)	6664/93 877 (7)	0.109
Thiazide diuretics (%)	6900/165 242 (4)	5606/94 446 (6)	<0.001
Aspirin (%)	173 064/192 911 (90)	96 895/109 272 (89)	<0.001
P2Y12 inhibitor (%)	178 504/194 359 (92)	99 048/109 954 (90)	<0.001
Statins (%)	162 253/193 665 (84)	87 218/109 592 (80)	<0.001
ACE inhibitors/ARB (%)	142 953/193 358 (74)	77 000/109 447 (70)	<0.001
Beta-blockers (%)	157 751/192 762 (82)	85 875/109 106 (79)	<0.001
Radionuclide study (%)	4307/166 725 (3)	2270/94 482 (2)	0.005
Exercise test (%)	7116/169 181 (4)	2662/96 305 (3)	<0.001
Coronary angiogram (%)	139 441/186 209 (75)	61 103/105 414 (58)	<0.001
Percutaneous coronary intervention (%)	74 139/151 965 (49)	28 466/81 747 (35)	<0.001
CABG surgery (%)	13 824/151 965 (9)	3695/81 747 (5)	<0.001
Revascularization (CABG surgery/PCI) (%)	87 963/151 965 (58)	32 161/81 747 (39)	<0.001
Death (%)	8978/194 805 (5)	7294/110 209 (7)	<0.001
Cardiac mortality (%)	7047/194 805 (4)	5681/110 209 (5)	<0.001
Reinfarction (%)	1601/181 953 (1)	950/102 847 (1)	0.233
Major bleeding (%)	2851/191 316 (1)	1924/108 375 (2)	<0.001
MACE (%)	10 248/194 805 (5)	7978/110 209 (7)	<0.001

IV, intravenous; MRA, mineralocorticoid receptor antagonist; ACE, angiotensin-converting-enzyme; ARB, angiotensin receptor blockers; CABG, coronary artery bypass graft; PCI, percutaneous coronary intervention; MACE, major adverse cardiovascular events.

MACE is defined as composite endpoint of in-hospital death and reinfarction.

### ESC ACVC quality indicators

Women less frequently received ICA within 73 hours’ time frame of admission (60% vs. 66%, *P* < 0.001). They were less frequently discharged on DAPT (82% vs. 84%, *P* < 0.001) and were less frequently discharged on a combination of DAPT and statin therapy (composite score) (68% vs. 73%, *P* < 0.001) (*Table 3*).

**Table 3:** Sex differences in ESC ACVC quality indicators in NSTEMI

Variables	Males (*n* = 194 805)	Females (*n* = 110 209)	*P*-value
Coronary angiography received within 72 h (%)	59 532/90 725 (66)	23 142/38 422 (60)	<0.001
LV function recorded in notes (%)	98 132/194 805 (50)	52 931/110 209 (48)	<0.001
Adequate P2Y_12_ inhibition on discharge (%)	178 504/194 359 (92)	99 048/109 954 (90)	<0.001
Fondaparinux or LMWH received (%)	144 811/168 187 (86)	82 395/95 808 (86)	0.471
DAPT received on discharge (%)	162 254/193 118 (84)	89 649/109 429 (82)	<0.001
High intensity statin on discharge^a^ (%)	162 253/193 665 (84)	87 218/109 592 (80)	<0.001
ACEi or ARB on discharge for those with moderate and severe LVSD (%)	30 472/39 547 (77)	15 329/20 149 (76)	0.008
Beta blocker on discharge for those for those with moderate and severe LVSD (%)	33 470/39 491 (85)	16 682/20 117 (83)	<0.001
Composite all/none score^b^ (%)	140 802/193 381 (73)	74 700/109 550 (68)	<0.001
Composite all/none score for those with moderate and severe LVSD (%)	28 720/39 573 (73)	14 115/20 140 (70)	<0.001

ESC, European Society of Cardiology; ACVC, Association for Acute Cardiovascular Care; GRACE, global registry of acute coronary events; CRUSADE, can rapid risk stratification of unstable angina patients suppress adverse outcomes with early implementation of the ACC/AHA guidelines; LV, left ventricle; EF, ejection fraction; LMWH, low molecular weight heparin; DAPT, dual antiplatelet therapy; ACEi/ARB, angiotensin converting enzyme inhibitor/angiotensin receptor blockers; LVSD, left ventricular systolic dysfunction; N/A, not available.

a MINAP does not record the specific type of statins, so ‘statin prescription’ was used as a surrogate for high intensity statin.

b Composite score of receipt of low dose aspirin, P2Y_12_ inhibition and statin.

### Multivariate analysis

Following adjustment for differences in age, ethnicity, serum creatinine, baseline comorbidities, LV systolic function, Killip classification, and cardiac arrest, women had significantly greater odds of in-hospital mortality (OR; 1.22, 95% CIs 1.17–1.28, *P* < 0.001) or MACE (OR; 1.18, 95% CIs 1.13–1.23, *P* < 0.001), which persisted when additionally adjusting for medication (in-hospital mortality OR; 1.12, 95% CIs 1.07–1.16, *P* < 0.001 and MACE OR; 1.10, 95% CIs 1.06–1.14, *P* < 0.001). When additionally adjusting for revascularization, there was no statistical significance in the odds of in-hospital mortality (OR; 0.99, 95% CIs 0.94–1.03, *P* = 0.52) or MACE (OR; 0.99, 95% CIs 0.94–1.05, *P* = 0.85) in women compared to men (*Table 4*).

**Table 4:** Adjusted outcomes for female versus male patients with NSTEMI

	Females (*n* = 110 209) (control group of males *n* = 194 805)							
	Model 1^a^		Model 2^b^		Model 3^c^		Model 4^d^	
Outcome variables	OR	*P*-value	OR	*P*-value	OR	*P*-value	OR	*P*-value
Primary outcomes								
MACE (in-hospital)	0.93 (0.90–0.96)	<0.001	1.18 (1.13–1.23)	<0.001	1.10 (1.06–1.14)	<0.001	0.99 (0.94–1.05)	0.847
Mortality (in-hospital)	0.92 (0.89–0.96)	<0.001	1.22 (1.17–1.28)	<0.001	1.12 (1.07–1.16)	<0.001	0.99 (0.94–1.03)	0.522
Secondary outcomes								
Cardiac mortality (in-hospital)	0.92 (0.89–0.96)	<0.001	1.26 (1.20–1.33)	<0.001	1.13 (1.08–1.19)	<0.001	1.07 (1.01–1.13)	0.020
Major bleeding (in-hospital)	1.02 (0.96–1.08)	0.601	1.05 (0.98–1.12	0.165	1.04 (0.98–1.11)	0.183	0.99 (0.93–1.06)	0.806

Hierarchical logistic regression model with females compared with reference group of males, with patients nested within hospitals.

a Model 1: Adjusted for age.

b Model 2: Model 1 +, ethnicity, creatinine, heart rate, blood pressure, history of acute myocardial infarction, history of stroke, history of peripheral vascular disease, Kilip classification, asthma or COPD, ejection fraction, cardiac arrest, smoking, hypertension, and hypercholesterolaemia.

c Model 3: Model 2 +, medication strategy (warfarin, LMWH, furosemide, fondaparinux, statins, P2Y12 inhibitors, aspirin, ACEi or ARB, unfractionated heparin, IV nitrate, aldosterone antagonist, thiazide diuretic, GPIIb/IIIa inhibitors and beta-blockers).

d Model 4: Model 3 + invasive coronary angiography and revascularization by PCI or CABG.

### Hospital-clustered analysis

**Figure 2:**
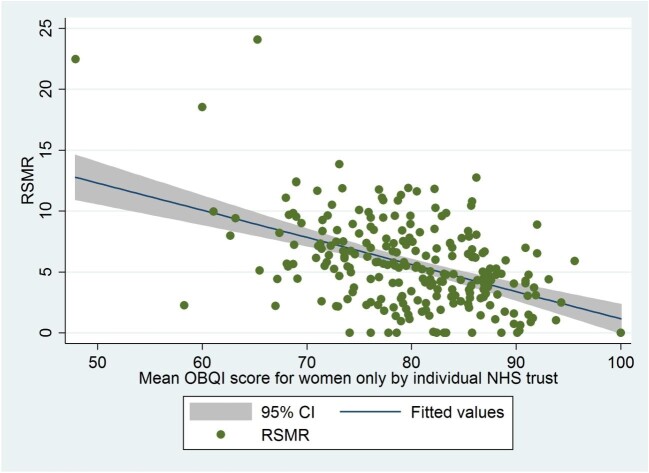
Risk standardized mortality rate (RSMR) for individual National Health Service (NHS) trusts patients plotted against mean Opportunity Based Quality Indicator Score (OBQI) for females. Coefficient −0.22, (95% CI; −0.278 to −0.168), *R*^2^ = 0.22, *P* < 0.001. RSMR, risk-standardized mortality rate. Adjusted for age, ethnicity, family history of coronary heart diseases, ischaemic ECG changes, history of heart failure, left ventricle systolic dysfunction, hypercholesterolaemia, history of myocardial infarction, history of cerebrovascular accident, history of peripheral vascular disease, hypertension, smoking, asthma/chronic obstructive pulmonary disease and admission under consultant cardiologist. Mortality refers to all-cause inpatient mortality only. Opportunity based QI [The score consisted of six evidence-based processes of care: the prescription of aspirin, thienopyridine inhibitor, β-blocker, angiotensin converting enzyme inhibitor (ACEi), HMG CoA reductase enzyme inhibitor (statin) and enrolment onto a cardiac rehabilitation programme at the time of discharge]. The OBCS reflects the number of care opportunities fulfilled at each hospital (numerator) divided by the number of opportunities to provide care (denominator). Excluded from both numerator and denominator were interventions that were contra-indicated, not applicable, not indicated in, or declined by, individual patients.

**Figure 3:**
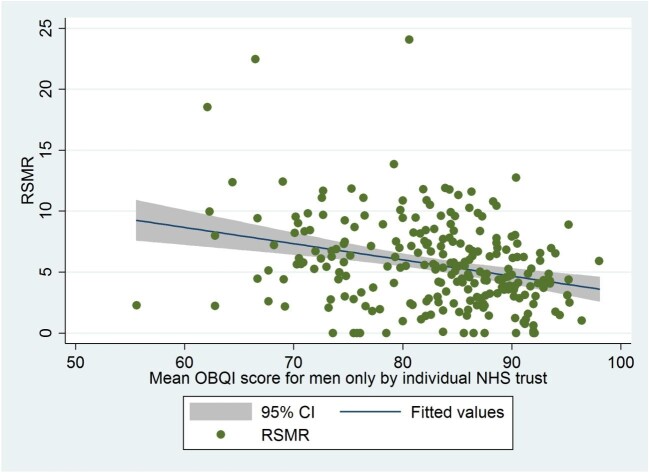
Risk standardized mortality rate (RSMR) for individual National Health Service) (NHS) trusts patients plotted against mean Opportunity Based Quality Indicator score (OBQI) for males. Coefficient −0.13, (95% CI; −0.190 to −0.076), *R*^2^ = 0.08, *P* < 0.001. Opportunity based QI [The score consisted of six evidence-based processes of care: the prescription of aspirin, thienopyridine inhibitor, β-blocker, angiotensin converting enzyme inhibitor (ACEi), HMG CoA reductase enzyme inhibitor (statin) and enrolment onto a cardiac rehabilitation programme at the time of discharge]. The OBCS reflects the number of care opportunities fulfilled at each hospital (numerator) divided by the number of opportunities to provide care (denominator). Excluded from both numerator and denominator were interventions that were contra-indicated, not applicable, not indicated in, or declined by, individual patients.

There is a positive correlation between the increasing proportion of women treated for NSTEMI in each individual hospital and RSMR [coefficient 0.034 (95% CIs; 0.024−0.438), *P* < 0.001, (*R*^2^ = 0.17)] (Supplementary material online, Figure S1). There is a stronger negative correlation between mean OBQI and RSMR in women [coefficient −0.22, (95% CI: −0.278 to −0.168), *R*^2^ = 0.22, *P* < 0.001] (*Figure 2*), than is seen in men [coefficient −0.13, (95% CI: −0.190 to −0.076), *R*^2^ = 0.08, *P* < 0.001] (*Figure 3*).

### Heat maps

**Figure 4:**
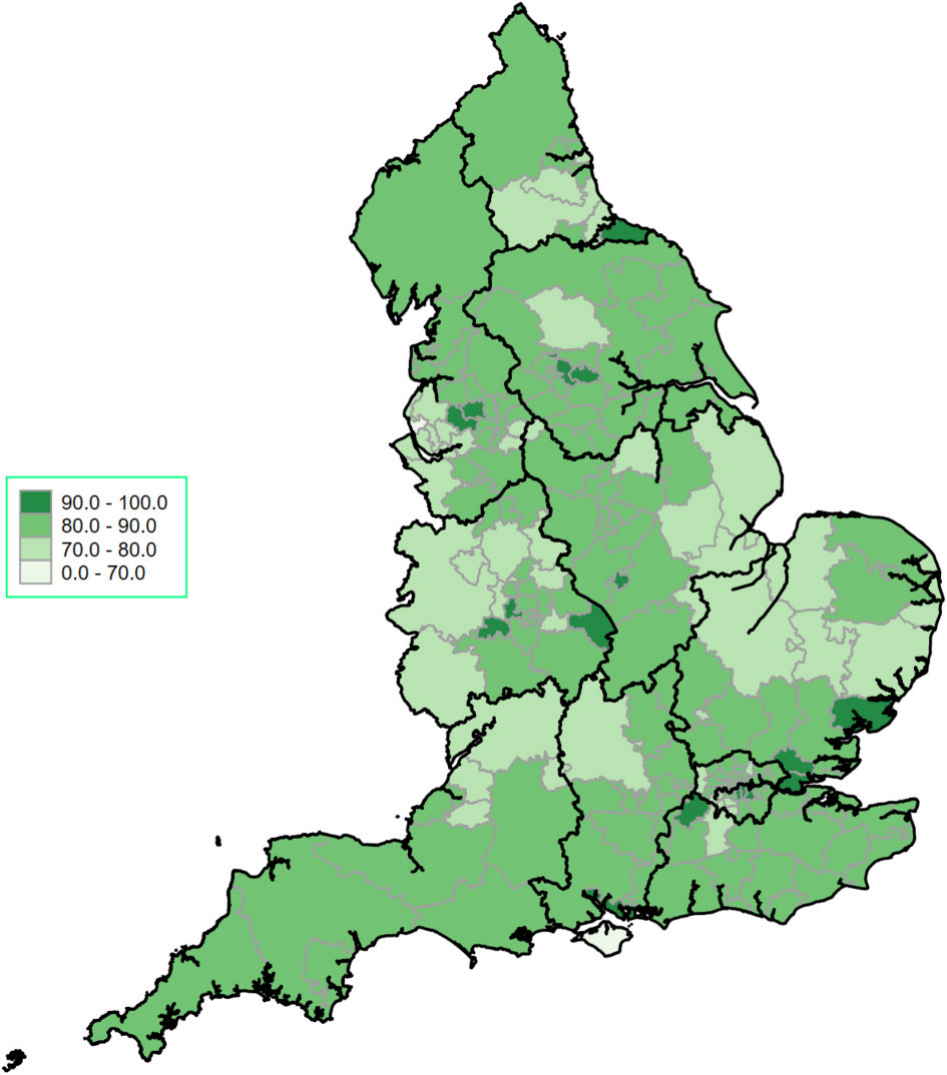
Heat map of UK Clinical Commissioning Groups (CCGs) according to mean Opportunity Based Quality Indicator (OBQI) score. Opportunity based QI (The score consisted of six evidence-based processes of care: the prescription of aspirin, thienopyridine inhibitor, β-blocker, angiotensin converting enzyme inhibitor (ACEi), HMG CoA reductase enzyme inhibitor (statin) and enrolment onto a cardiac rehabilitation programme at the time of discharge). The OBCS reflects the number of care opportunities fulfilled at each hospital (numerator) divided by the number of opportunities to provide care (denominator). Excluded from both numerator and denominator were interventions that were contra-indicated, not applicable, not indicated in, or declined by, individual patients.

**Figure 5:**
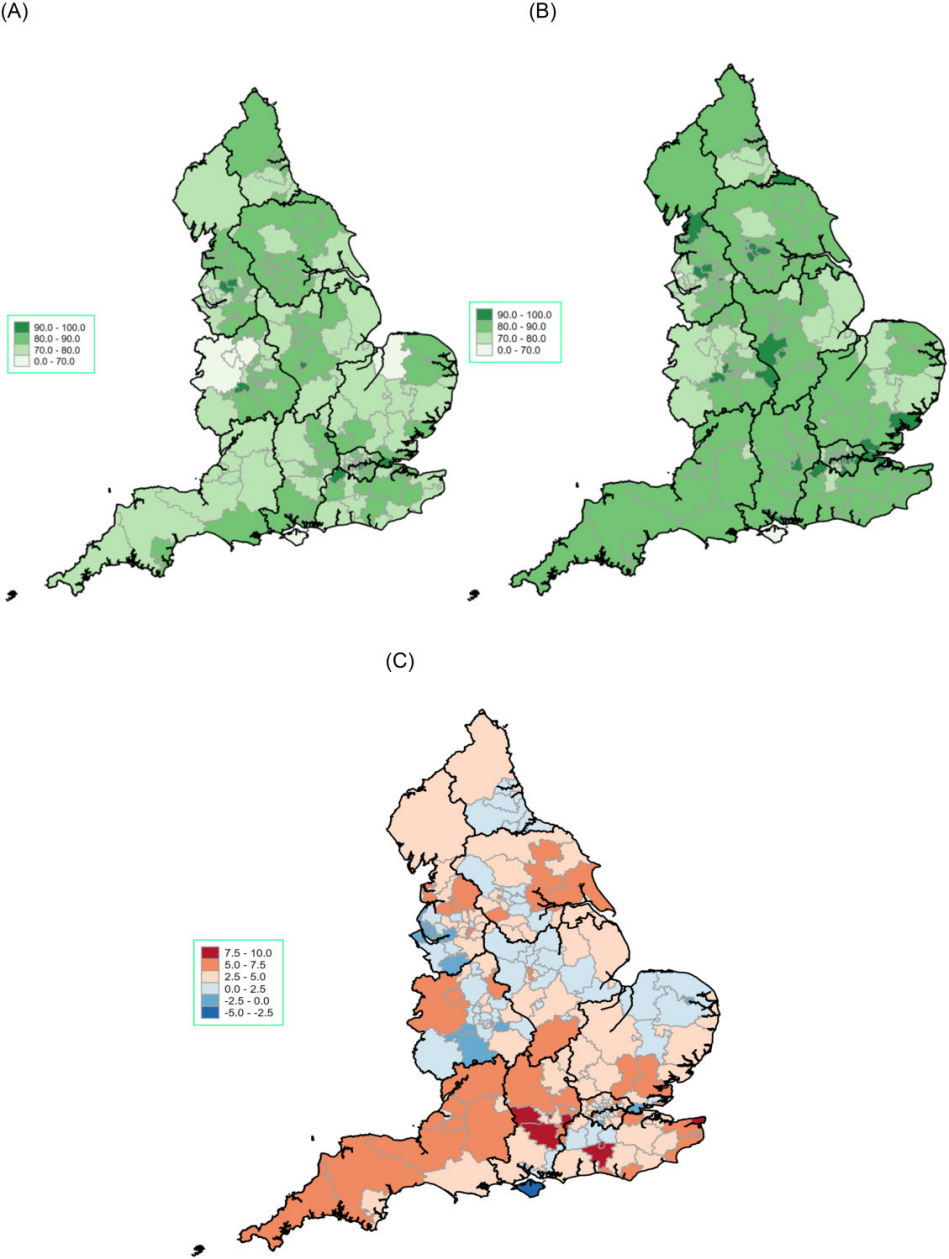
Heat maps of UK Clinical Commissioning Groups according to mean opportunity based quality indicator score according to sex. (A) Heat map of UK Clinical Commissioning Groups (CCGs) according to mean Opportunity Based Quality Indicator (OBQI) score for females. (B) Heat map of UK Clinical Commissioning Groups (CCGs) according to mean Opportunity Based Quality Indicator (OBQI) score for males. (C) Heat map of UK Clinical Commissioning Groups (CCGs) according to difference in mean Opportunity Based Quality Indicator (OBQI) score between males and females (male score minus female score). The mean difference in OBQI is displayed, calculated by male score minus female score. Opportunity based QI [The score consisted of six evidence-based processes of care: the prescription of aspirin, thienopyridine inhibitor, β-blocker, angiotensin converting enzyme inhibitor (ACEi), HMG CoA reductase enzyme inhibitor (statin) and enrolment onto a cardiac rehabilitation programme at the time of discharge]. The OBCS reflects the number of care opportunities fulfilled at each hospital (numerator) divided by the number of opportunities to provide care (denominator). Excluded from both numerator and denominator were interventions that were contra-indicated, not applicable, not indicated in, or declined by, individual patients.

*Figure 4* is a ‘heat map’ of the UK CCGs according to the mean OBQI score. *Figure 5* is a collection of ‘heat maps’ of the mean OBQI score for each UK 2021 CCG according to sex. This shows a marked regional variation in the mean OBQI score throughout the UK, but notable are the lower mean scores in Wales, Shropshire, the Northwest, and the East of England. *Figure 5**(A)* shows that for women only, the mean OBQI is lower throughout the UK but particularly poor in the same regions (Wales, Shropshire, Northwest, and East of England). The equivalent ‘heat map’ for men (*Figure 5**(B)*) shows a higher mean OBQI score throughout the UK, with particularly high mean OBQI scores in the Northeast, East Midlands, and Southeast.

### Supplementary analysis

*Table 5* shows the adjusted mortality rates for the sex and OBQI care categories. A lower proportion of women received ‘good’ or ‘fair’ care compared to their male counterparts. ‘Poor’ and ‘excellent’ care was similar between the sexes. *Table 6* shows the mean OBQI scores for men and women in all regions of the UK. There are no regions where care is deemed ‘excellent’ for women. Comparatively, care in south-east London is rated as ‘excellent’ for men. Furthermore, there are no geographical regions where care is superior for women compared to men.

**Table 5:** Adjusted mortality rates for gender and OBQI care category combinations

OBQI score category and gender combination	Adjusted in-hospital mortality (with 95% CI)	*P*-value
‘Poor’ care and male	12.0% (11.0–12.4%)	<0.001
‘Poor’ care and female	12.0% (11.0–12.4%)	<0.001
‘Fair’ care and male	5.0% (4.6–5.3%)	<0.001
‘Fair’ care and female	4.8% (4.5–5.2%)	<0.001
‘Good’ care and male	2.3% (2.1–2.4%)	<0.001
‘Good’ care and female	2.1% (1.9–2.3%)	<0.001
‘Excellent’ care and male	0.8% (0.7–0.9%)	<0.001
‘Excellent care’ and female	0.8% (0.6–0.9%)	<0.001

Opportunity based QI [The score consisted of 6 evidence-based processes of care: the prescription of aspirin, thienopyridine inhibitor, β-blocker, angiotensin converting enzyme inhibitor (ACEi), HMG CoA reductase enzyme inhibitor (statin) and enrolment onto a cardiac rehabilitation programme at the time of discharge]. The OBCS reflects the number of care opportunities fulfilled at each hospital (numerator) divided by the number of opportunities to provide care (denominator). Excluded from both numerator and denominator were interventions that were contra-indicated, not applicable, not indicated in, or declined by, individual patients.

OBQI score ordered into four categories, ‘Excellent’, ‘Good’, ‘Fair’, and ‘Poor’. Excellent refers to an OBQI score of ≥90 and ⇐100, Good a score of ≥80 and <90, Fair ≥70 and <80, and Poor <70.

Mortality rates adjusted for age, ethnicity, family history of coronary heart diseases, ischaemic ECG changes, history of heart failure, left ventricle systolic dysfunction, hypercholesterolemia, history of myocardial infarction, history of cerebrovascular accident, history of peripheral vascular disease, hypertension, smoking, asthma/chronic obstructive pulmonary disease and admission under consultant cardiologist.

**Table 6:** Modelled adjusted lives saved over study period if care in females is improved to the standard of males, according to OBQI score category

Region	Female population	Female mean OBQI score	Male mean OBQI score	Female OBQI score classification	Male OBQI score classification	Estimated number of lives that would be saved (95% CIs)
East of England	13 201/35 522 (37%)	78.3	82.1	Fair	Good	356 (330–396)
South East	8626/23 180 (37%)	80.5	85.8	Good	Good	N/A
South Central	5186/15 308 (34%)	83.6	87.9	Good	Good	N/A
South West	9953/27 839 (36%)	77.9	83.8	Fair	Good	269 (249–299)
Yorkshire and the Humber	13 394/36 676 (37%)	82.9	86.3	Good	Good	N/A
North West	19 550/50 524 (39%)	79.0	81.6	Fair	Good	528 (489–587)
North East	7429/20 162 (37%)	81.4	84.1	Good	Good	N/A
West Midlands	8584/25 419 (34%)	79.7	82.6	Fair	Good	232 (215–258)
East Midlands	6396/18 592 (34%)	81.2	83.4	Good	Good	173 (160–192)
Wales	4974/13 479 (37%)	73.3	75.9	Fair	Fair	N/A
Northern Ireland	1600/4995 (32%)	84.1	84.5	Good	Good	N/A
North central London	1515/4752 (32%)	82.0	86.4	Good	Good	N/A
North East London	2495/7624 (33%)	78.6	83.3	Fair	Good	67 (62–75)
North West London	2345/6511 (36%)	74.9	77.9	Fair	Fair	N/A
South East London	2409/7108 (34%)	86.0	90.5	Good	Excellent	34 (29–39)
South West London	2552/7323 (35%)	81.0	83.7	Fair	Good	69 (64–77)

Adjusted mortality for comparison of ‘Good’ care in women with ‘Fair’ care in women with 95% CIs: 2.7% (2.5–3.0%), *P* < 0.001. Adjusted mortality for comparison of ‘Excellent’ care in women with ‘Good’ care in women with 95% CIs: 1.4% (1.2–1.6%), *P* < 0.001. OBQI score is ordered into four categories: ‘Excellent’, ‘Good’, ‘Fair’, and ‘Poor’. ‘Excellent’ refers to an OBQI score of 100, ‘Good’ refers to a score of ≤100 and ≥83, ‘Fair’ refers to a score of ≥67 and <83, and ‘Poor’ refers to a score of <67. Mortality rates are adjusted for age, ethnicity, family history of coronary heart diseases, ischaemic ECG changes, history of heart failure, left ventricle systolic dysfunction, hypercholesterolaemia, history of myocardial infarction, history of cerebrovascular accident, history of peripheral vascular disease, hypertension, smoking, asthma/chronic obstructive pulmonary disease, and admission under a consultant cardiologist. Lives saved are estimated for regions where women’s care by mean OBQI was in a lower category than that for men, where the number of women included in the study period is multiplied by the difference in mortality rates.

## Discussion

Our nationwide analysis of over 300 000 NSTEMI patients in the UK revealed a number of important findings. First, there are marked sex-based differences in the quality of care received by patients admitted with a NSTEMI, with women less frequently treated on a dedicated cardiology ward or admitted under a cardiologist, less frequently treated with GDMT, and less frequently in receipt of coronary revascularization. Second, we report marked regional variation in the quality of care received by women, with no geographical areas where the care for women was noted to be superior to that of men. Third, the difference in care between men and women varies significantly by both the individual region and hospital. Fourth, in our hospital-clustered analysis, we show a positive correlation between the RSMR and the increasing proportion of women treated for NSTEMI. Finally, there is a stronger negative correlation between the mean opportunity based quality indicator and RSMR in women than is seen in men, suggesting that there may be greater potential gains in improving the quality of care in women than in men.

Prior studies examining sex disparities in AMI have several important limitations and contextual differences. Several studies have been undertaken in the USA, a multipayer health care system in which the health insurance of patients may affect the quality of care they receive, both with respect to primary prevention and acute illnesses. Furthermore, previous studies have predominantly focused on AMI collectively, or just STEMI. Given the less algorithmic management of NSTEMI, larger inequalities in the quality of care are more likely. Our study is the first to assess sex disparities in NSTEMI on a nationwide scale with granular interhospital variability.

Our heat maps of the UK CCG's according to the mean OBQI score show that the overall care is inferior for women compared to men. The overwhelming majority of geographical areas for women are rated as ‘fair’ whereas for men, the overwhelming majority are rated as ‘good’. There are also more areas rated as ‘poor’ and fewer areas rated as ‘excellent’ for women in comparison to men. Geography has previously been shown to be a key determinant of health, and location has been shown to affect access to treatment as well as the baseline cardiometabolic risk factor profile of an individual.^16^ Baum et al. showed in their retrospective cohort study of over 5 million participants that in the United States (US), moving from a 10th to a 90th percentile zip code for a given outcome was associated with a significantly increased prevalence of uncontrolled blood pressure, diabetes, and obesity.^16^ Tu et al., as part of their retrospective cohort study involving 5.5 million patients in Ontario, Canada, showed that access to healthcare was better in geographic regions where mortality was lower. Specifically, residents in those areas received physician services more often, were more likely to hit targets for hypertension and hypercholesterolaemia, and were screened more often.^17^

Our scatter plots show a negative correlation of the RSMR and mean OBQI, with significantly lower RSMR associated with improved OBQI scores. This relationship is far stronger for women compared to men. Although the UK is a publicly funded healthcare system, where you would expect a degree of homogeneity in AMI management between hospitals, *Chung et al.* showed that there is greater variation in 30-day mortality post-AMI in the UK compared to Sweden, which they attribute to greater heterogeneity in the prescription of GDMT amongst UK hospitals.^18^ It is not necessarily clear whether this is reflective of poorer practice in the UK or whether this is reflective of excellence in the Swedish system. *Gale et al.* have shown this hospital-wide variation in practice across the UK using MINAP data, with numerous hospitals shown to be below control limits for ‘door to needle time’ and for aspirin and beta-blocker usage post AMI.^19^ Interestingly, the observed variation between hospitals may not all be attributable to in-hospital processes of care, with *Asaria et al.* recently suggesting that a significant proportion of inter-hospital variation in mortality is due to regional myocardial event rates,^20^ influenced by risk factors such as smoking, hypertension, and hypercholesterolaemia. The strong relationship between the RSMR and OBQI scores in women compared to men suggests that there are significant gains to be made with respect to improving the mortality of women presenting with NSTEMI by improving the quality of care that they receive in the hospital.

It is important to note that the poorer quality of care for women is not just confined to the UK and US, with registry data from Portugal suggesting performance in AMI management with regard to time to ICA and GDMT prescription (using the ESC QIs) is poorer in women,^21^ and data from Australia suggesting that even after adjusting for comorbidities, women are less likely to undergo coronary intervention.^22^ Our results showed that when adjusting for baseline characteristics or when adjusting for baseline characteristics and management strategy (inclusive of medications), women had worse primary outcomes of in-hospital mortality and MACE and worse secondary outcomes of cardiac mortality. When additionally adjusting for invasive strategy and revascularization, there were no significant differences in primary or secondary outcomes between men and women, highlighting the importance of not just guideline-directed medical treatment but the importance of an invasive strategy in reducing the disparities in key outcomes such as mortality between men and women.

Our study has important clinical implications for practice. Following the passage of the 2022 Health and Care Act in the UK, significant reforms have shifted the way the English health and care system is organized.^23^ The formation of integrated care systems, which are partnerships that bring together NHS organizations, local authorities, and others to take collective responsibility for planning services, improving health, and reducing inequalities across geographical areas, has resulted in 42 autonomous geographical entities in the UK. Whilst traditionally in many countries, financing and autonomy of healthcare systems have been a top-down approach, with regional variation less pronounced, advanced healthcare systems, including those in the US, differ significantly based on state, with funding and arrangements of healthcare services differing widely between the different states. With an increasing population and increasing geographical disparities in healthcare, it would be expected that over time, the regional autonomy of healthcare budgets will become more pronounced. Our study shows wide geographic variation in the quality of care received for women, with specific geographic areas having a worse mean OBQI (Wales, Shropshire, Northwest, and East of England), with women being more adversely affected in these regions. With the aim of these reforms to uniform the quality of care between geographic areas, our study highlights the significant geographic disparities in care for NSTEMI patients, particularly in areas where significant ‘levelling up’ is required for women.

## Strengths of study

There are a number of strengths to this study. Our analysis represents the largest study to date that looks at differences in NSTEMI patients by sex in the UK and the first to look at interhospital variability in quality of care in such granular detail. The MINAP database encapsulates an almost complete record of NSTEMI patients admitted in the UK and represents one of the largest national real-world databases of this cohort of patients in the world, including those who are high-risk and have multiple comorbid illnesses, such that they are either not included or under-represented in clinical trials.

## Limitations of study

There are important limitations to observational studies of this type. The MINAP data registry shares the weaknesses of other national registries, including self-reporting of adverse events with no external validation. Although the MINAP dataset included important clinical and demographic variables of interest, there are limitations to the data collected. For instance, the database does not capture frailty score, severity of CAD, psychosocial risk factors, access to use of healthcare, rationale for specific medications, or an exhaustive comorbidity list. Furthermore, the database does not capture markers of inflammation, biomarkers, LDL-c levels, or less common risk factors such as malignancy, lipoprotein(a) or clonal haematopoiesis of indeterminate potential. Furthermore, inherent to large registries such as MINAP is the issue of missing data, we have attempted to mitigate against this by using MICE. We acknowledge that there is no external adjudication of diagnosis and endpoints of cases in our registry and that there may be a range of practices across UK hospitals, including the usage of different troponin assays and changes in clinical practice regarding GDMT, cardiac rehabilitation referral, and invasive investigations over what is a relatively long study period; therefore, our results must be interpreted in the context of this. For example, in a small minority of multimorbid patients, there may be a misclassification of type-two myocardial infarction for type-one myocardial infarction, where our quality indicators may not be relevant to this population. For our OBQI score calculations, patients for which pharmacotherapy agents were contraindicated or not-applicable are excluded from the numerator and denominator; however, it is possible that in the data entry, there may be patients for which certain agents were legitimately contraindicated/not-applicable that may be recorded as being omitted if the reason for this was not clearly documented or communicated.

Our study used MINAP registry data from 2010 to 2017, whereas our heat maps are based upon 2021 CCG boundaries, due to the lack of availability of editable maps from that range of years. A small number of CCGs were combined into larger groups over this time, which required manual reclassification and recalculation. Finally, in our RSMR scatter plots, there are several outlier values, which reflect hospitals with small numbers of patients; thus, the individual rates in these hospitals should be interpreted with caution.

## Conclusion

Our study showed that women presenting with NSTEMI in the UK are typically older and increasingly comorbid. Women received a poorer quality of care, with lower rates of ICA, PCI, and GDMT prescriptions when compared with men. There was wide geographical variation in the mean OBQI scores according to sex. There were no areas in the UK where women had superior care to men. The strong relationship between the RSMR and OBQI scores in women compared to men suggests that there are significant gains to be made with respect to improving the mortality of women presenting with NSTEMI by improving the quality of care that they receive in the hospital. Further work should focus on reducing the geographical disparities in access to high-quality NSTEMI care according to sex.

## Supplementary Material

qcae011_Supplemental_File

## Data Availability

The data underlying this article were provided by the National Institute for Cardiovascular Outcomes Research (NICOR). Data will be shared on request to corresponding author with permission of NICOR.
