# Supplementary material for: Sex-based analysis of NSTEMI processes of care and outcomes by hospital: a nationwide cohort study
Source: Eur Heart J Qual Care Clin Outcomes. 2024 Feb 6;10(8):750–62. doi: 10.1093/ehjqcco/qcae011 (PMC11656063; doi:10.1093/ehjqcco/qcae011)
Supplement: qcae011_Supplemental_File [file qcae011_supplemental_file.docx]

**Supplementary Figure 1: RSMR for individual NHS trusts patients plotted against proportion of patients that are female**

**
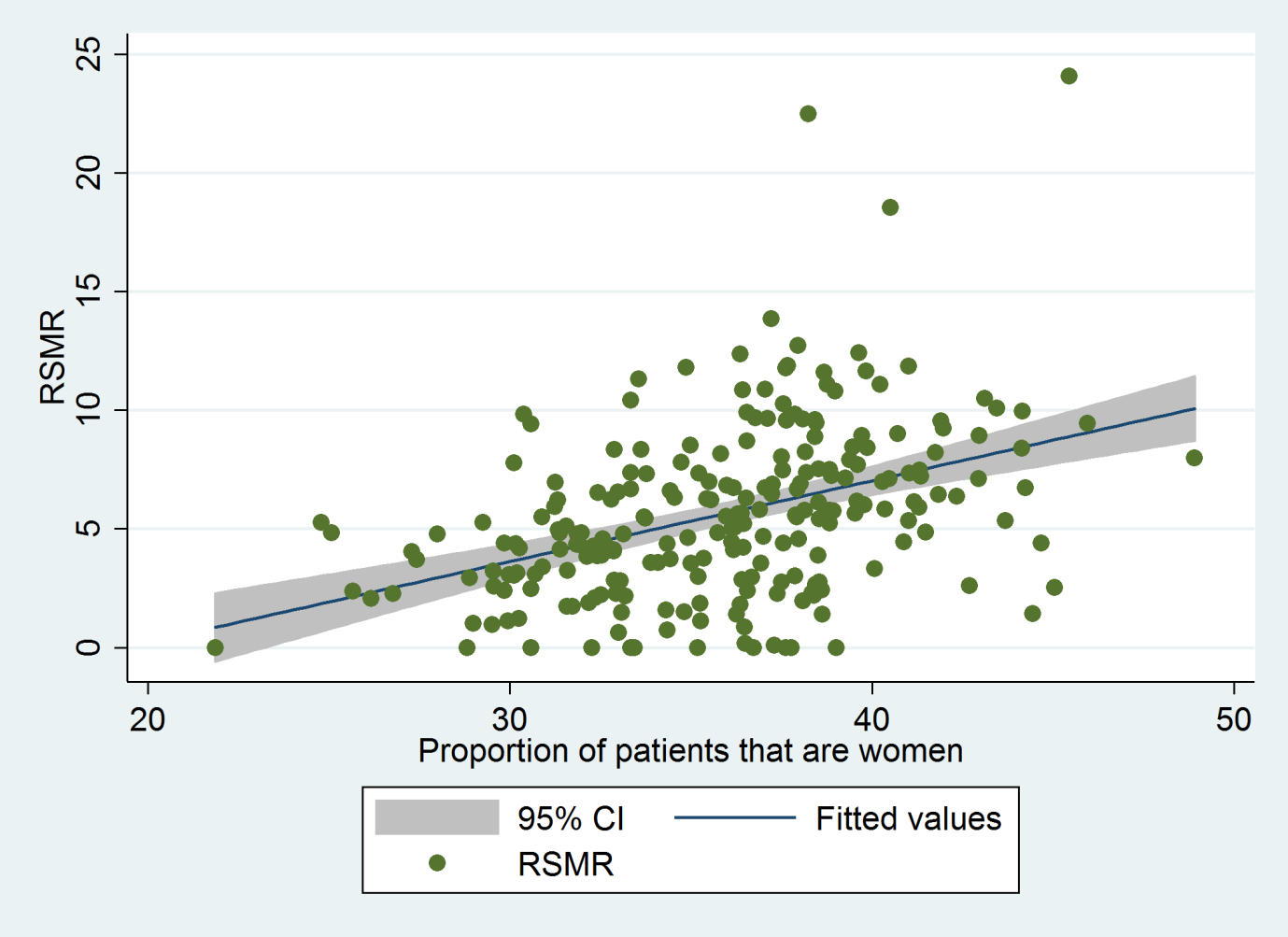
**

(Coefficient 0.034 (95% CI; .024 -.438, P<0.001, (R^2^=0.17)). CI, confidence interval.

RSMR; risk-standardized mortality rate. Adjusted for age, ethnicity, family history of coronary heart diseases, ischaemic ECG changes, history of heart failure, left ventricle systolic dysfunction, hypercholesterolaemia, history of myocardial infarction, history of cerebrovascular accident, history of peripheral vascular disease, hypertension, smoking, asthma/chronic obstructive pulmonary disease, and admission under consultant cardiologist. Mortality refers to all-cause inpatient mortality only.
